# An approach for prevention planning based on the prevalence and comorbidity of neurodevelopmental disorders in 6-year-old children receiving primary care consultations on the island of Menorca

**DOI:** 10.1186/s12887-023-03844-2

**Published:** 2023-01-20

**Authors:** Lorena Francés, Jessica Caules, Antoni Ruiz, Catalina Virgínia Soler, Amaia Hervás, Alberto Fernández, Alberto Rodríguez-Quiroga, Javier Quintero

**Affiliations:** 1 Child and Adolescent Psychiatrist, Menorca (Balearic Islands, Spain), Av. Del Metge Camps 20, 07740 Es Mercadal, Balearic Islands Spain; 2 Arrels Institute, Ciutadella, Balearic Islands Spain; 3 University of Barcelona, Barcelona, Spain; 4 Dalt Sant Joan Center (Mahón), Servei de Salut de les Illes Balears, Mahón, Illes Balears Spain; 5 Child–Adolescent Mental Health Unit at the Mutua Terrasa University Hospital, Barcelona, Catalonia Spain; 6 the Autonomous University of Barcelona, Barcelona, Catalonia Spain; 7 Saint George Hospital in London, London, UK; 8 Child–Adolescent Psychiatry at Maudsley Hospital, London, UK; 9 Department of Legal Medicine, Psychiatry and Pathology at the Complutense University of Madrid, Madrid, Spain; 10 Hospital Universitario Infanta Leonor, Madrid, Spain; 11 Psychiatry Service of the Infanta Leonor Hospital, Madrid, Spain; 12 Psychiatry Department of the Complutense University of Madrid, Leading Expert in Child and Adolescent Psychiatry, Madrid, Spain

**Keywords:** Prevalence, Comorbidity, Neurodevelopmental disorders, DSM-5, Childhood

## Abstract

**Background:**

Few studies have estimated the real prevalence of neurodevelopmental disorders according to the Diagnostic and Statistical Manual of Mental Disorders, 5th Edition (DSM-5) in Spain and worldwide. However, there are disparate prevalence figures. We consider research in this field essential to improve early detection, secondary prevention, and health planning.

**Methods:**

The Minikid ADHD and TICS-Mini International Neuropsychiatric Interview for Children and Adolescents, the Autism Spectrum Quotient (Children’s version, AQ- Child) and a protocol of general medical questions were administered for screening purposes. The PROLEXIA battery for children aged from 4 to 6 years was used for direct assessments. Parents provided information on emotional, medical, and school aspects. The final population evaluated using these tools consisted of 291 6-year-old subjects.

**Results:**

The overall risk of presenting with a neurodevelopmental disorder was 55.4%. A 23.4% risk of presenting with attention-deficit/hyperactivity disorder (ADHD) in any modality (inattentive, hyperactive-impulsive and combined), a 2.8% risk of developing autism spectrum disorder (ASD), a 30.6% risk of presenting with a learning disorder with reading difficulties, a 5.5% risk of tics and a 22.5% risk of language problems (incomprehensible language or minor language problems) were detected in the sample. The most common combination of disorders was learning and language difficulties, accounting for 6.9% of the sample. The second most frequent combination was the presence of learning and language difficulties and ADHD, accounting for 4.5% of the sample.

**Conclusions:**

The prevalence of risks detected in our sample seems to be consistent with national and international studies. A significant proportion of our sample had never been previously diagnosed (85%), so early detection programs are recommended.

## Introduction

According to the latest revised version of the Statistical Manual of Mental Disorders [1], which coincides with the DSM-5 [2], neurodevelopmental disorders (NDs) are those that include a clinical manifestation in almost all developmental domains. These manifestations include intellectual disability (ID), as well as those that affect more specific domains, such as attention-deficit/hyperactivity disorder (ADHD), autism spectrum disorder (ASD), communication disorders (CDs), specific learning disorders (SLDs, including difficulties in reading, writing and mathematics), and motor skill disorders (MDs, such as Tics, Tourette’s syndrome and stereotypic disorders), among others [3].

NDs usually begin in childhood, although most of them are chronic and persist for life. A new approach is committed to the inclusion of NDs within a heterogeneous and dimensional group, leaving behind the categorical classifications of the DSM-4th edition [4] and the International Statistical Classification of Diseases and Related Health Problems (ICD) [5]. The new edition of the ICD (ICD-11) unifies its criteria with those of the DSM-5 (2013). Finally, the revised DSM-5 (i.e., DSM-5-TR) was recently published in 2022.

To our knowledge, there are only a few studies in the scientific literature that measure the prevalence of NDs in minors according to DSM-5 criteria (2013). The prevalence rates reported in 2022 were as follows: ID, 0.63%; ADHD, 5–11%; ASD, 0.70–3%; SLDs, 3–10%; CDs, 1–3.42%; and MDs, 0.76–17% [3, 6–11].

Among the available literature, prevalence studies and meta-analyses are the most common. The prevalence rates of the most common NDs were estimated as follows: ADHD, 7.9–9.5% [12, 13]; SLDs, 0.7–2.2% [12, 14, 15]; SLDs (including developmental dyslexia [DD]), 1.2–24% [16, 17]; and MDs, 1.4–19% [18, 19]. Furthermore, reported prevalence rates for various disorders within the same study did not include comorbidity rates between disorders [10].

In the United States, according to data published by the National Center for Health Statistics (NCHS) in 2015, it is estimated that 15% of children between the ages of 3 and 17 years are affected by NDs [20].

In a previous systematic review by our research team [3], we found that the global prevalence rate of NDs fluctuates globally between 4.70% in Scotland [8], 55.5% in Norway [9], and 88.50% in Japan [10].

These variations depended on methodological aspects, such as estimation procedures and sociocontextual phenomena. The criteria used by the different studies varied, and the processes used to measure the indicators were often not explicitly stated. In addition, it should be considered that the validity and reliability of the assessment instruments used were not explicitly mentioned and, in many cases, were nonexistent. In our study, we used a diagnostic approach to consider the problems of measurement and validity with respect to the data collection techniques used not only in our study but also in general.

There was also little direct evaluation and, consequently, little diagnostic certainty regarding the clinical populations in these studies. Furthermore, the studies often did not consider the complexity and comorbidities of the disorders; instead, symptoms or risks tended to be analyzed individually. Secondary sources are important as complementary resources for diagnosis, but prevalence studies with direct sources are lacking. Among the few studies where symptoms were directly assessed, in the study of Catalonia [7] and Norway [9], clinical diagnostic measures and questionnaires were used to assess symptoms and were completed by teachers. The Japan study [10] used surveys and questionnaires completed by parents and teachers. Most prevalence studies used indirect estimates such as health database records, which are likely to be less accurate. For example, not explicitly stating the overlap of comorbidities and results could be misleading and lead to overestimating the risk. However, we believe that this is a complex aspect from an empirical point of view. In our study, we have used the added criterion of diagnostic risk in the tests administered; specifically, we report the number of diagnoses for each participant.

NDs are usually underdiagnosed [21]. Therefore, children who have not been diagnosed are more likely to suffer from emotional and behavioral problems, low self-esteem, lower-than-expected academic performance, difficulties in social relationships, unemployment, delinquent behavior and functional impairment [22, 23]. The implementation of early detection and early intervention programs is essential [24].

NDs usually present as homotypic comorbidities, and it is rare that they occur in isolation. Despite this, there is a large body of literature on specific disorders, and these disorders have rarely been evaluated as a whole.

Multimorbidity among individuals with NDs is the norm, as determined in Japan [10], low-resource countries [6], Scotland [8], Spain [11], and Norway [9]. The prevalence of NDs seems to remain stable over time in different cultures, ages, ethnic groups [25] socioeconomic strata, types of communities (rural or urban), and religions [26]. Gender differences in NDs are consistent, with males being most affected by general psychiatric psychopathology, as reflected in studies in Scotland [8] and Denmark [27].

Males are more affected by NDs; 66.3% of the children included in a cross-sectional study in Norway [9] were male, and in a sequential cross-sectional study in Japan [28], a male:female ratio of 2.2:1 was reported. With regard to ADHD, male:female ratios of 4:1 and 2:1 were determined in a systematic review and meta-analysis in Spain [29, 30], generally corresponding with the reported ratios (3-2:1) in the systematic reviews by Sayal et al. (2018) and Faraone et al. (2021). Finally, a male:female ratio of 4.5:1 was reported in children with ASD in a retrospective analytical cohort study [31].

Considering the prevalence variations found in the different studies analyzed worldwide, we considered it necessary to carry out more studies in nonclinical samples and with direct evaluations that better reflect the reality of the population. In Catalonia [7] and the USA [32], a study was conducted with a school sample; in Galicia [11], Catalonia [31], Norway [9] and Brazil [33], a study was conducted with a clinical sample of children receiving specialized mental health services.

In countries with low socioeconomic resources [6], such as China [34] and Japan [10], a sample of the general population (rural and urban) was obtained. For this reason, we decided to conduct this study in a primary care sample, which we thought would more accurately reflect prevalence risk approximations than clinical samples. The age of 6 years was selected to insist on early detection and to demonstrate the possibility of providing an intervention through secondary prevention. These interventions can be performed at early ages when neuronal plasticity is still present, even if only the most severe forms of learning problems are usually detected at 6 years of age. Due to the heterogeneity of these disorders, the choice of 6 years of age limits our ability to diagnose the most severe cases of ASD or ID that would be detected before 3 years of age. Even so, we have observed an underdiagnosis of the more subtle forms of ASD in children with higher IQs. This is the first study in a school-age population where an exhaustive and direct assessment was carried out by professionals trained in neurodevelopment.

This is the first study of its type that has been carried out in a nonclinical population on the island of Menorca and with direct observations of the participants.

The general objective of the study was to obtain evidence on the prevalence and comorbidity of NDs to establish the fundamental elements for good health planning based on secondary prevention and early detection of subtle symptoms, which may go unnoticed if not explored in primary care services.

The specific objectives were as follows:

- to estimate the prevalence of NDs on the island of Menorca based on standardized tests and interviews with parents.
- to establish the territorial differentiation of each disorder, considering its prevalence.
- to determine the comorbidity of these disorders on the island.
- to predict the presence of a comorbidity based on the NDs analyzed and the sociodemographic characteristics analyzed and considered.

Finally, this study measured the risks of presenting any ND according to the DSM-5 (that is, ADHD, ASD, SLDs, MDs and CDs) and their possible comorbidities. ID, learning disabilities with difficulties in writing and mathematics and motor coordination problems were not assessed due to time and cost limitations.

There are numerous studies in the literature on the possible association between the environment and the development of NDs. Multiple and varied environmental factors have been studied (biological, social and economic factors). We could say that they comprise a broad spectrum of environmental pollutants [35, 36], pre/perinatal risk factors [37, 38] unhealthy lifestyle habits and disadvantaged environments (social exclusion, poverty, low purchasing power) [39, 40]. All these factors could act at the epigenetic level, modifying gene expression and favoring the development of a given condition.

## Material and methods

### Participants and procedure

**Fig. 1:**
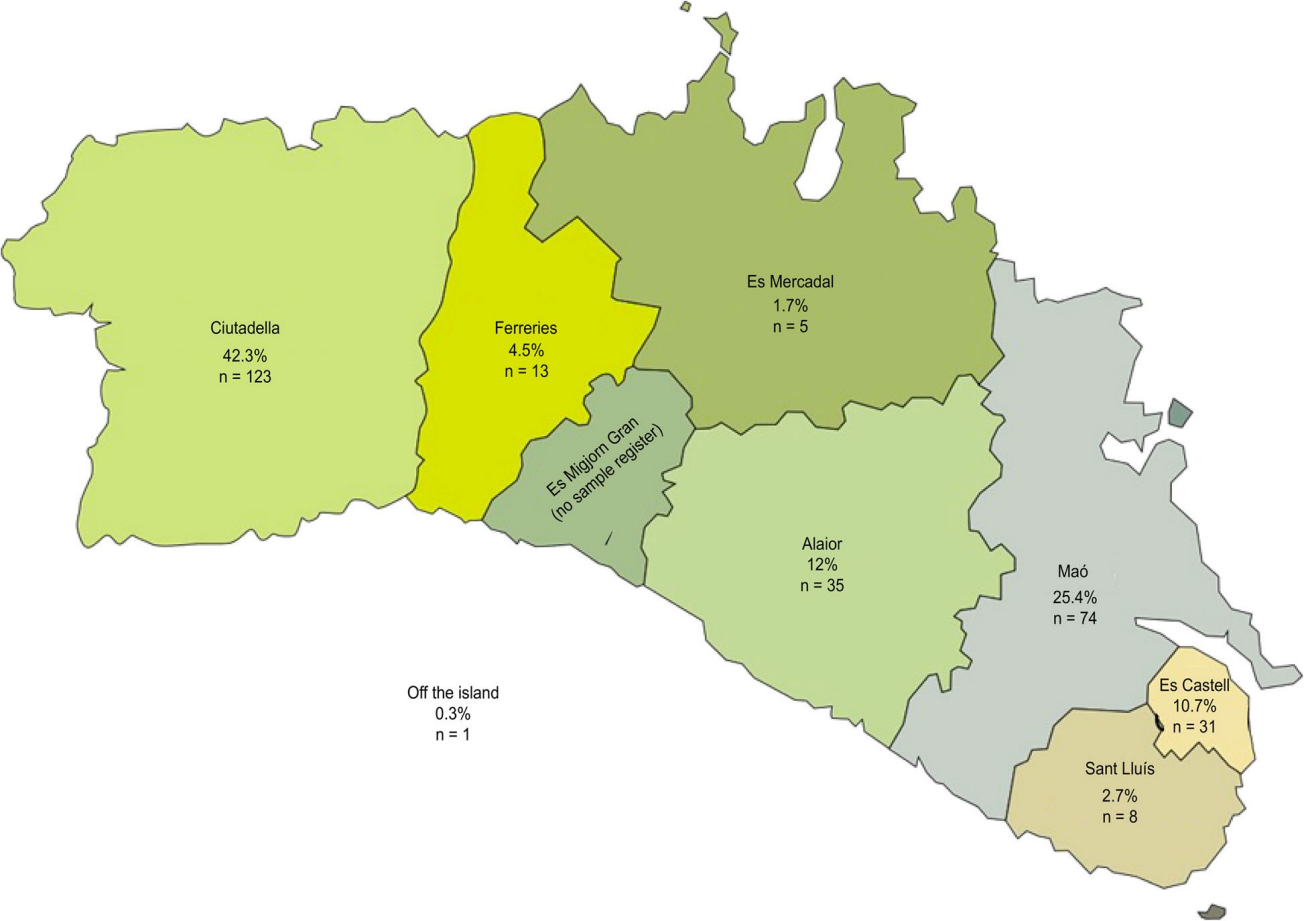
Distribution of the participating population

The sample was drawn from the Menorcan population. All the health centers of Menorca participated: Health Centers of Mahón (Dalt Sant Joan), CS Es Castell, CS Ferreries, CS Es Banyer, CS Mercadal, CS Sant Lluís and Ciutadella (Canal Salat). Figure 1 shows the proportion of participation by municipality and the number of participating subjects. The collaboration rates were higher in Canal Salat (Ciutadella) (75%), CS Es Castell (70%), Dalt Sant Joan (Mahón) (50%), and Es Banyer (45%). The lowest responders were CS Ferreries (10%), Es Mercadal (20%) and Sant Lluís (15%).

The sample size for an estimated maximum ND prevalence of 25% on the island of Menorca to achieve a precision of +/− 5% with a 95% confidence interval and *p* = 0.25 was 289 subjects. It was calculated according to the 2021 Registry (which refers to 01–01-21); the 5-year-old population (born in 2015) included 850 subjects, and the 6-year-old population (born in 2014) included 821 subjects. Therefore, to obtain a sample with adequate representativeness for this type of study (sampling errors from 3 to 5%), 289 subjects would be necessary.

After approval from the ethics committee of the Balearic Islands (CEIB) in December 2020, the sample was collected consecutively by pediatricians and nurses during the months of January, February and March 2021, which was the time necessary to obtain a representative sample size of 289 children. Parents of children who attended their 6-year checkup were invited to participate in the study, and subjects who agreed to participate were recruited. The investigator and collaborators evaluated the parents who agreed to collaborate in the study after they signed the informed consent form. Security measures were taken to ensure the confidentiality of the data.

A total of 345 subjects were initially recruited through their pediatrician. Thirty-eight subjects were lost in this recruitment phase due to personal reasons and travel difficulties, so 307 children were ultimately evaluated in the first phase of the study. Of these 307 participants, the sample was reduced to 289, with 18 losses due to incomplete assessments, a lack of information and dropouts.

The study was carried out 1 year after the declaration of the COVID-19 pandemic situation, adopting all the required safety measures and with masks worn during the evaluation, a fact that should be taken into account when interpreting the results.

The parents collected the questionnaires they had previously filled out from their pediatrician’s office and gave them to the investigators on the day of the direct evaluation with the child.

### Inclusion and exclusion criteria

Children who attended the consultation of the Child-Adolescent Health Program of the Primary Care Consultations in Menorca for the 6-year follow-up visit, which could be carried out from 2 months before turning 6 years old to 1 month before turning 7 years old, were included.

Children diagnosed with NDs that had been detected at previous ages were not excluded, and reports were accepted if they came from accredited entities with specialized professionals.

All children under 5 years and 11 months and over 7 years of age at the time of the evaluation were excluded.

### Study description

**Fig. 2:**
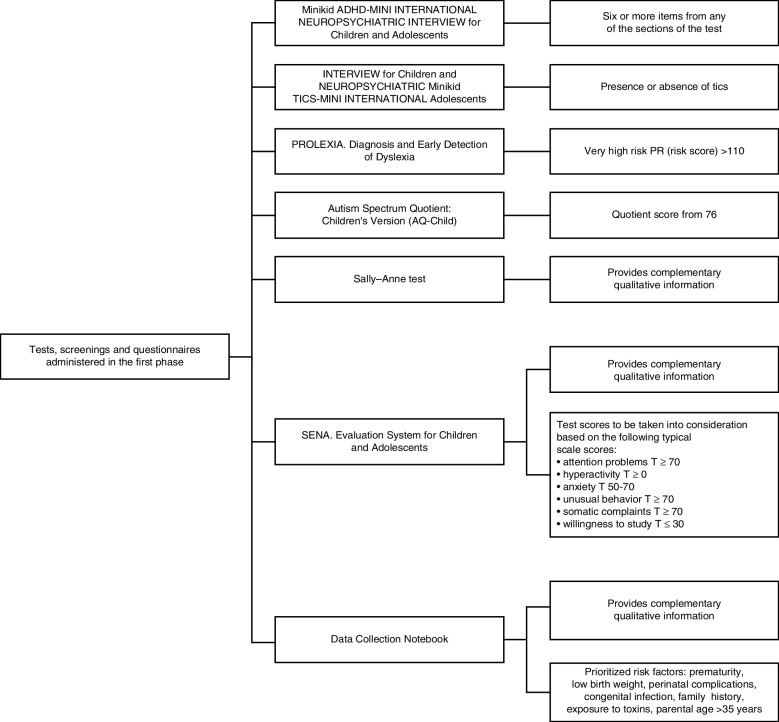
The specific instruments used. The figure shows more screening tools/tests than those described in the text; in the article, we only describe those we used to measure risks for neurodevelopmental disorders

The sample was collected from routine well child consultations in primary care services according to the Child-Adolescent Health Program of the Balearic Islands [41]. Subsequently, the families who decided to participate in the study were summoned by professionals trained in neurodevelopment and underwent an exhaustive general evaluation (of the child and their parents separately) with different instruments that evaluated different areas and warning signs. Clinical data were collected through a Data Collection Notebook that included risk factors associated with NDs in the literature, such as prematurity, low birth weight, pre- and perinatal infections, medical history, parental age, and exposure to toxic substances. Instruments were included to evaluate alarm signals (shown in Fig. 2) that were used as screening tools to measure the risk of presenting NDs. Thus, children were classified as being or not being at risk of presenting NDs. During the assessment of the 289 subjects and families, durations of approximately 20–30 minutes and 30–40 minutes were used for direct observation of the minor and their parents, respectively.

### Measures

#### The Minikid ADHD and TICS- Mini international neuropsychiatric interview for children and adolescents [42]

As one author noted, “the MINI-KID generates reliable and valid psychiatric diagnoses for children and adolescents” [42]. It is a brief structured diagnostic interview for diagnosing DSM-IV and ICD-10 psychiatric disorders in children and adolescents. We decided to use this interview to perform a quick and sensitive screening of the risk of presenting ADHD and tic disorders. As a limitation, this interview is based on the previous diagnostic manual; however, we decided to use it because of its speed of administration and because the diagnostic criteria between the current and previous manual do not differ significantly for initial screening. It determines the risk of presenting ADHD and tics in any of its presentations.

#### The autism spectrum quotient (children’s version, AQ-child [43]

Indeed, “this is an instrument that aims to quantify autistic traits in children aged 4–11 years” [43]. The AQ-Child is a 50-question questionnaire that is answered by parents and used to detect autistic traits. We used the Spanish version from the Autism Research Center (Cambridge), which is designed to be administered to parents. A score above or equal to 75 points determines the risk of ASD. The psychometric validity and reliability procedure for this instrument is not reported.

#### The PROLEXIA battery for the early detection and differential diagnosis of dyslexia [44]

The PROLEXIA tool is used for the early detection of potential cases of dyslexia. The duration of the test is 30 minutes, and the correction is performed online. Since this is a new test, there is no literature to date, which urges us to carry out more studies to demonstrate its replicability and validity. Children are classified as having very low, low, moderate, high, moderate, high, and very high risk of presenting dyslexia based on the obtained scores. For a risk score (RP) of 57 or higher, moderate risk is present and therefore considered indicative of the presence of dyslexia according to the test score [44]. We used this tool because it is a measure in the Spanish language, and there are few instruments that measure the early risk of dyslexia.

### Data collection notebook

This questionnaire, prepared by our research team, collects data on sociodemographic variables, the medical and mental health histories of both the mother and child, lifestyle habits and general medical information. It consists of 120 questions.

*The Sally and Annie Test (Baron-Cohen S.* et al.*, 1985) and the System of Evaluation of Children and Adolescents (SENA) for parents* [45] were used to obtain information that is complementary to the rest of the instruments presented here and to obtain greater diagnostic validity.

### Statistical analysis

First, the variables used in this study are presented in Table 1, which shows the dimensions analyzed and their corresponding variables and indicators, as well as the level of measurement used for each of them.

**Table 1:** Study analysis variables

Dimension	Variables		Measure: dichotomous categorial
Global aspects ND diagnosis	Comorbidity		**Yes – No (1.0):** Aspects of risk diagnosed in the tests. At least two tests indicate the presence of the disorder.
	Presence of ND risk		**Yes – No (1.0):** At least one test shows a score indicating the presence of the disorder.
Sociodemographic and clinical variables	Sex	Male Female	**Yes – No (1.0)**
	Course	Kindergarten, 5 years Primary	**Yes – No (1.0)**
	Territory	Ciutadella Alajor-EsMerca-Ferre Mao EsCaste_S. Lluis	**Yes – No (1.0)**
	Economic resources	Low Medium High	Classification based on parents’ perceptual responses. **Yes – No (1.0)**
	Premature birth		**Yes – No (1.0)**
	Breastfeeding		**Yes – No (1.0)**
	Low birth weight		**Yes – No (1.0):** 1 = With 2500 g. or less
	Congenital infection (Preg.50)		**Yes – No (1.0)**
	Maternal age > 45 years		**Yes – No (1.0)**
	Edad parent > 45 years		**Yes – No (1.0)**
	Toxicity in pregnancy		**Yes – No (1.0)** With tobacco, alcohol, or hashish consumption
	Eutochic childbirth		**Yes – No (1.0)**
	Instrumental birth		**Yes – No (1.0)**
	Cesarean delivery		**Yes – No (1.0)**
Diagnostic tests	PROLEXIA		**Yes – No (1.0):** 1 = Very high and moderate scores were grouped as a risk of PROLEXIA
	ADHD-MiniKid		**Yes – No (1.0)** 1 = 6 or more risk items
	A.Q.C.		**Yes – No (1.0)** 1 = Score of 75 or higher
	TICS-Minikid		**Yes – No (1.0)** 1 = Presence of tics

*ND* Neurodevelopmental disorder, *PROLEXIA* PROLEXIA Battery for early detection and differential diagnosis of dyslexia, *ADHD-MiniKid* Minikid ADHD-Mini International Neuropsychiatric Interview for Children and Adolescents, *AQC* Autism Spectrum Quotient (Children’s version, AQ-Child); *TICS-Minikid* Minikid TICS-Mini International Neuropsychiatric Interview for Children and Adolescents

The test scores were indicative and recorded in an Excel database. Regarding the analysis of the information collected, SPSS (Statistical package for the Social Sciences, v. 27), [46] was used, performing a univariate analysis (percentages of the variables) and an inferential analysis for comparing two categorical variables (Chi-square test). Finally, a predictive analysis of the variable “comorbidity” (yes or no) was performed using bivariate logistic regression.

## Results

### Sociodemographic characteristics of the study population

Our population sample was collected using records of children who were affiliated with social security, so we could say that 100% of the sample was collected from the public health system database (Ib-salut). It should be noted that 5% of children undergo concomitant private and public follow-up, i.e., they are affiliated with social security and undergo closer follow-up in private services, which is a common practice on the island. Data on ethnic and racial diversity were not collected.

The final sample population comprised 289 subjects. Figure 1 shows the percentages of the participating population in each municipality.

Of this population, 46.7% were girls (*n* = 136), and 53.3% were boys (*n* = 155) according to consecutive random selection. These children attended a total of 54 different schools on the island. Eighty-five percent of the children had not been previously diagnosed.

The perceived level of economic resources was predominantly medium (89.7%), determined using a subjective assessment completed by parents of their perceived socioeconomic status. The parents predominantly had a university education.

### Prevalence of NDs and comorbidity

Table 2 shows the prevalence of NDs according to sociodemographic characteristics. Table 3 shows the prevalence of comorbidity according to demographic characteristics and screening test results.

**Table 2:** Prevalence of NDs according to demographic characteristics

Sociodemographic characteristics		Total, N (%)	Developments			
			Presence of NDs		Absence of NDs	
			N (unweighted %)	Weighted % (95% CI)	N (unweighted %)	Weighted % (95% CI)
Sex	Boy	155 (53.60)	101 (62.70)	0.62 (0.54–0.70)	54 (42.20)	0.42 (0.33–0.51)
	Girl	134 (46.40)	60 (37.30)	0.373 (0.29–0.45)	74 (57.80)	0.57 (0.48–0.66)
Course	Q5	120 (41.59)	66 (41.00)	0.42 (0.34–0.50)	54 (42.30)	0.42 (0.34–0.52)
	Primary	162 (56.10)	90 (55.90)	0.57 (0.49–0.65)	72 (56.30)	0.57 (0.48–0.65)
Place of residence	Ciutadella	122 (42.20)	66 (41.00)	0.41 (0.33–0.49)	56 (43.80)	0.43 (0.35–0.52)
	Alajor-Merca-Ferre	52 (18.00)	29 (18.00)	0.18 (0.12–0.25)	23 (18.00)	0.18 (0.11–0.25)
	Mao	74 (25.60)	48 (28.6)	0.28 (0.22–0.36)	28 (21.90)	0.21 (0.15–0.30)
	EsCaste-St. Lluis	39 (13.30)	18 (11.2)	0.11 (0.06–0.17)	21 (16.40)	0.16 (0.10–0.24)
Economic resources	Low	114 (39.40)	74 (46.00)	0.47 (0.39–0.55)	40 (31.30)	0.31 (0.23–0.40)
	Media	27 (9.30)	14 (8.70)	0.08 (0.05–0.14)	13 (10.20)	0.10 (0.05–0.17)
	High	142 (49.10)	69 (42.90)	0.43 (0.36–0.52)	73 (57.00)	0.57 (0.48–0.66)
Premature birth	Yes	26 (9.00)	15 (9.30)	0.09 (0.05–0.15)	11 (8.60)	0.08 (0.04–0.15)
Breastfeeding	Yes	221 (76.50)	117 (72.70)	0.73 (0.65–0.79)	104 (81.30)	0.81 (0.73–0.87)
Low birth weight	Yes	34 (11.80)	17 (10.60)	0.10 (0.06–0.16)	17 (13.30)	0.13 (0.08–0.20)
Congenital infection	Yes	6 (2.10)	4 (2.50)	0.02 (0.00–0.06)	2 (1.60)	0.01 (0.00–0.05)
Maternal age > 35 years	Yes	83 (28.70)	40 (24.80)	0.25 (0.18–0.32)	43 (33.60)	0.33 (0.25–0.42)
Paternal age > 45 years	Yes	11 (3.80)	5 (3.10)	0.03 (0.01–0.07)	6 (4.70)	0.04 (0.01–0.10)
Toxicity in pregnancy	Yes	35 (12.10)	22 (13.70)	0.13 (0.08–0.20)	13 (10.20)	0.10 (0.05–0.16)
Childbirth type	Eutocic	165 (57.10)	88 (54.70)	0.54 (0.46–0.62)	77 (60.20)	0.60 (0.51–0.68)
	Instrumental	29 (10.0)	20 (12.40)	0.12 (0.07–0.18)	9 (7.00)	0.07 (0.03–0.12)
	Cesarean section	94 (32.50)	51 (31.70)	0.31 (0.24–0.39)	43 (33.60)	0.33 (0.25–0.42)
Total		289 (100)	161 (55.7 0)	0.55 (0.49–0.61)	128 (44.3 0)	0.44 (0.38–0.52)

*95% CI* Confidence interval: binomial success rate for a sample (Clopper-Pearson), *ND* Neurodevelopmental disorder, *Q5* Kindergarten, 5 years

**Table 3:** Prevalence of comorbidity according to demographic characteristics and screening test results. Estimates and (95% CIs)

Sociodemographic characteristics		Total, N (%)	Comorbidity			
			Presence of comorbidity		Absence of comorbidity	
			N (unweighted %)	Weighted % (95% CI)	N (unweighted %)	Weighted % (95% CI)
Sex	Boy	155 (53.60)	40 (70.20)	0.70 (0.56–0.81)	115 (49.60)	0.49 (0.43–0.56)
	Girl	134 (46.40)	17 (29.80)	0.29 (0.18–0.43)	117 (50.40)	0.50 (0.43–0.57)
Course	Q5	120 (41.59)	20 (35.10)	0.35 (0.22–0.48)	100 (43.10)	0.44 (0.37–0.51)
	Primary	162 (56.10)	37 (64.90)	0.64 (0.51–0.77)	125 (53.90)	0.55 (0.48–0.62)
Place of residence	Ciutadella	122 (42.20)	23 (4 0.40)	0.40 (0.27–0.54)	99 (42.70)	0.43 (0.36–0.49)
	Alajor-Merca-Ferre	52 (18. 0)	12 (21.10)	0.21 (0.11–0.33)	40 (17.20)	0.17 (0.12–0.22)
	Mao	74 (25.60)	18 (31.60)	0.31 (0.19–0.45)	56 (24.10)	0.24 (0.18–0.30)
	EsCaste-St. Lluis	39 (13.30)	4 (7. 00)	0.07 (0.01–0.17)	35 (15.10)	0.15 (0.10–0.20)
Economic resources	Low	114 (39.40)	36 (63.20)	0.64 (0.50–0.76)	78 (33.60)	0.34 (0.28–0.40)
	Media	27 (9.30)	4 (7. 00)	0.07 (0.02–0.17)	23 (9 0.90)	0.10 (0.06–0.14)
	High	142 (49.10)	16 (28.10)	0.28 (0.17–0.42)	126 (54.30)	0.55 (0.48–0.62)
Premature birth	Yes	26 (9.0 0)	9 (15.80)	0.16 (0.07–0.29)	17 (7.30)	0.07 (0.04–0.11)
Breastfeeding	Yes	221 (76.50)	41 (71.90)	0.73 (0.59–0.84)	180 (77.60)	0.77 (0.71–0.82)
Low birth weight	Yes	34 (11.80)	8 (14. 00)	0.14 (0.06–0.26)	26 (11.20)	0.11 (0.07–0.16)
Congenital infection	Yes	6 (2.10)	3 (50.30)	0.05 (0.01–0.14)	3 (1.30)	0.01 (0.0 3–0.03)
Pregnant age > 35 years	Yes	83 (28.70)	16 (28.10)	0.28 (0.17–0.42)	67 (28.90)	0.29 (0.23–0.35)
Parental age > 45 years	Yes	11 (30.80)	3 (5.30)	0.05 (0.01–0.15)	8 (3.40)	0.03 (0.01–0.07)
Toxicity in pregnancy	Yes	35 (12.10)	7 (12.30)	0.12 (0.05–0.24)	28 (12.10)	0.12 (0.08–0.17)
Childbirth type	Eutocic	165 (57.10)	30 (52.60)	0.52 (0.39–0.66)	135 (58.20)	0.58 (0.51–0.64)
	Instrumental	29 (10.0)	6 (10.50)	0.10 (0.04–0.21)	23 (9.90)	0.09 (0.06–0.14)
	Cesarean section	94 (32.50)	20 (35.10)	0.35 (0.22–0.48)	74 (31.90)	0.31 (0.25–0.38)
High PROLEXIA	Yes	89 (30.80)	48 (84.20)	0.84 (0.72–0.92)	41 (17.70)	0.17 (0.13–0.23)
TDAH-Minikid	Yes	68 (23.50)	35 (61.40)	0.61 (0.47–0.74)	33 (14.20)	0.14 (0.1 0–0.19
A.Q.C.	Yes	8 (2.80)	4 (7.00)	0.07 (0.01–0.17)	4 (1.70)	0.01 (0.0 5–0.04)
TICS-Minikid	Yes	16 (50.50)	9 (15.80)	0.15 (0.07–0.27)	7 (3.00)	0.03 (0.01–0.06)
Total		289 (100)	57 (19.70)	0.197 (0.15–0.24)	232 (8 0.30)	0.80 (0.75–0.84)

*95% CI* Confidence interval: binomial success rate for a sample (Clopper-Pearson), *Q5* Kindergarten, 5 years, *PROLEXIA* PROLEXIA Battery for the early detection and differential diagnosis of dyslexia, *ADHD-MiniKid* Minikid ADHD-Mini International Neuropsychiatric Interview for Children and Adolescents, *AQC* Autism Spectrum Quotient (Children’s version, AQ-Child), *TICS-Minikid* Minikid TICS-Mini International Neuropsychiatric Interview for Children and Adolescents

The school years that were evaluated were p5 (40.9%) and the 1st year of primary school (56.3%). No statistically significant differences were found according to the school year in any measured risk; therefore, it could be concluded that school year was not an interfering factor.

As measured by the PROLEXIA tool, a very high risk of presenting a learning disorder with reading difficulties was established for 8.6% of the population; a high risk was established for 13.4% of the population and a moderate risk was established for 8.6% of the population, for a total risk percentage of 30.6%.

A risk of presenting ADHD in any modality (inattentive, hyperactive-impulsive and combined) of 23.4% was established according to the semistructured Mini International Neuropsychiatric Interview for Children and Adolescents (MINI-KID) ADHD interview.

A 2.8% risk of developing ASD was calculated according to the Autism Spectrum Quotient Children’s version (AQC).

A 5.5% risk of presenting any alteration due to tics was observed according to the MINI-KID Tics section. Using information extracted from the data collection notebook, in terms of psychomotor skills, 2.7% of the sample had difficulty going up/down stairs, 16.5% had difficulty tying their shoelaces, 7.9% had tics at some point in their lives, 3.1% had stereotypies and 0.3% had mannerisms, according to the clinical assessment of the investigator.

Considering the language level, alterations (incomprehensible language or minor language problems) were detected in 22.5% of the sample; this information was obtained from the clinical assessment of the investigator, the medical history and previous reports that described the difficulty or diagnosis, if any.

In this screening, it could not be determined whether there were learning disorders with writing and math difficulties, ID or motor coordination problems, although it is assumed that people with ID will have learning problems.

The risks of suffering from any neurodevelopmental difficulty (dyslexia, ADHD, language, ASD and tics) were estimated and combined, with an overall risk of having one or more NDs of 55.4%.

The most common comorbidities found were learning and language difficulties in 6.9% of the sample. The second most frequent comorbidity was the presence of difficulties in learning, language problems and ADHD (4.5%). The risk of comorbid NDs was calculated by combining the individual risks of any given ND.

### Differences by gender

Regarding learning difficulties, boys were more affected (53.6%, 55 boys) than girls (46.4%, 33 girls), with these differences being statistically significant (χ2 (1) = 4.461; *p* < 0.05).

Tics exclusively affected boys with a ratio of 16:0, with these differences being statistically significant according to sex (χ2 (1) = 14.643; *p* < 0.05).

Statistical differences were found according to sex for the presence of incomprehensible language or any type of minor alteration in language in early childhood. A total of 53.6% of the boys presented with incomprehensible language compared to 46.4% of the girls (χ2 (1) = 6.095; *p* < 0.05). Statistically significant differences were also determined according to sex for minor language alterations (χ2 (1) = 5.288; *p* < 0.05), with girls and boys presenting a risk of 46.4 and 53.6%, respectively.

Regarding ADHD, boys had a higher risk of comorbid ADHD (girls 26.5% vs. boys 73.7%) and the hyperactive-impulsive modality than girls (girls 39.3% vs. boys 60.7%), except in the inattentive modality (girls 9.7% vs. boys 5.2%). These differences were not statistically significant.

Regarding the risk of ASD, the boy:girl ratio was 7:1. No statistically significant differences were found according to gender.

### Relationship with ND risk

In our work, we included factors considered to be risk factors in the literature, and we added others for study, including sports practice, the consumption of new technologies, breastfeeding, type of childbirth, adherence to the Mediterranean diet, and educational and socioeconomic levels, among others. In general, children from disadvantaged families had a higher risk of suffering from one or more NDs (χ^2^_(2)_ = 19,728; *p* < 0.05).

### Relationship with comorbidity

We have evidenced statistically significant differences between the presence of comorbid NDs and its association with low socioeconomic resources (χ^2^_(2)_ = 16,901; *p* < 0,01) and prematurity (χ^2^_(1)_ = 4376; *p* < 0,05). The practice of sports was significantly associated with a lower presence of ND comorbidities (χ^2^_(1)_ = 7139; *p* < 0,01). In the Sally and Annie test, we found statistically significant differences (χ^2^_(1)_ = 6213; *p* < 0,05) between comorbidity and test alterations.

### Relationship with ASD

The risk of ASD did not show statistical significance for any of the variables studied.

### Relationship with language impairment

Low socioeconomic resources showed a statistically significant relationship with the presence of language impairment (χ^2^_(2)_ = 11,616; *p* < 0,01), the presence of prematurity (χ^2^_(1)_ = 4239; *p* < 0,05) and impairment shown on the Sally and Annie test (χ^2^_(1)_ = 10,756; *p* < 0,01).

### Relationship with dyslexia risk

The risk of dyslexia did not show statistical significance in any of the variables studied.

### Relationship with ADHD

Low economic resources were statistically significantly related to a greater presence of ADHD (χ^2^_(2)_ = 11,709; *p* < 0,05), and statistical significance was not obtained for the other variables studied (prematurity, diet, breastfeeding, low birth weight, sports practice or parental education level).

### Relationship with tics

The presence of breastfeeding was related to a lower risk of presenting tics, and this relationship was statistically significant (χ^2^_(1)_ = 3983; *p* < 0,05). The rest of the variables studied did not show significant differences.

### Predictors of comorbid NDs

A binary logistic regression model (5-step LR method) was established for the variable “comorbid NDs” to detect elements that could exert a predictive effect on this variable. In the predictive model of “Risk of having a comorbidity”, a Nagelkerke corrected R-squared value of 0.51 and a Cox and Snell R square value of 0.312 were obtained, with a prognostic probability of 85% of the sample. The best predictor found in the analysis corresponds to presenting high and very high risks of dyslexia according to the PROLEXIA battery and MINIKID TICS positivity, with PROLEXIA showing the highest predictive power (versus MINIKID TICS), as shown in Table 4.

**Table 4:** Comorbidity predictors among NDs and sociodemographic variables

		Variable in the equation							
		Coefficient “B”	Standard error (SE)	Wald	df	***p*** value	OR “Exp(B)”	95% CI for OR “EXP(B)”	
								Lower	Superior
Step 1	PROLEXIA _YES (Very High. High and Moderate)_NO	3.279	0.429	58.568	1	0.000	26.559	11.467	61.512
	Constant	−3.045	0.362	70.782	1	0.000	0.048		
Step 2	PROLEXIA_YES (Very High. High and Moderate)_NO	5.133	1.039	24.413	1	0.000	169.595	22.133	1299.512
	TDAH_MINIKID	4.396	1.036	18.005	1	0.000	81.117	10.649	617.916
	Constant	−5.724	1.040	30.265	1	0.000	0.003		
Step 3	PROLEXIA_YES (Very High. High and Moderate)_NO	21.324	2898.930	.000	1	0.994	1,823,623,128.658	0.000	.
	TDAH_MINIKID	20.259	2898.930	.000	1	0.994	628,835,561.606	0.000	.
	MINIKID_TICS	20.408	2898.930	.000	1	0.994	729,449,251.463	0.000	.
	Constant	−22.017	2898.930	.000	1	0.994	0.000		
Step 4	PROLEXIA_ YES (Very High. High and Moderate)_NO	3.629	0.508	50.993	1	0.000	37.691	13.919	102.064
	MINIKID_TICS	3.140	0.797	15.542	1	0.000	23.114	4.851	110.141
	Constant	−3.532	0.455	60.276	1	0.000	0.029		

Dependent variable: comorbidity; Independent variables: sociodemographic, clinical and psychometric variables

*PROLEXIA* PROLEXIA Battery for the early detection and differential diagnosis of dyslexia, *ADHD-MiniKid* Minikid ADHD-Mini International Neuropsychiatric Interview for Children and Adolescents, *AQC* Autism Spectrum Quotient (Children’s version, AQ-Child), *TICS-Minikid* Minikid TICS-Mini International Neuropsychiatric Interview for Children and Adolescents

It seems that the PROLEXIA tool allows, in a certain way and with a certain probability, the identification of other types of NDs associated with at least one disorder.

Furthermore, taking into account the sociodemographic variables used, the best predictors of the presence or absence of an ND, as shown in Table 5, are being male and having low declared economic resources.

**Table 5:** Prediction of the presence of NDs according to sociodemographic variables

		Variables in the equation							
		Coefficient “B”	Standard error (SE)	Wald	df	***p*** value	OR “Exp(B)”	95% CI for OR “EXP(B)”	
								Lower	Upper
Step 1^to^	MALE	0.757	0.258	8.619	1	0.003	2.131	1.286	3.533
	Constant	−0.211	0.188	1.258	1	0.262	0.810		
Step 2^b^	MALE	0.697	0.262	7.085	1	0.008	2.007	1.202	3.353
	Economic resources LOW	0.706	0.272	6.743	1	0.009	2.025	1.189	3.449
	Constant	−0.444	0.211	4.420	1	0.036	0.642		

Dependent variable: NDs; Independent variables: sociodemographic and clinical variables

## Discussion

Our work closely reflects and replicates prevalence results obtained in previous studies, and the risks of comorbidities and sex differences are consistent with previous findings. The measured risks of presenting with ADHD, ASD, dyslexia, language alterations or tics are close to those reported in the literature.

Sex (i.e., being male) and socioeconomic factors significantly influence the expression of certain conditions, such as major and minor language impairments and the presence of tics or hyperactivity, as previously demonstrated. As we have studied, there is a much higher risk of presenting with one or more NDs in disadvantaged environments, and there is a large body of literature in this regard. This was not observed for the risk of dyslexia, since it does not seem to be affected by year of schooling, socioeconomic factors, or other medical factors. This could increase the effect of genetics for future studies of this disorder; along the same lines, the genetic predisposition of ADHD [47], ASD [48] and language disorders [49] is also well known. Pennington and Lefly (2001) [50] demonstrate in their research that a total of 34% of their sample was at high risk for dyslexia. In the study, risk percentages were based on genetics and the inheritance of dyslexia in biological parents [50]. This percentage is similar to the that obtained in our research, with a total of 30.8%.

It is important to assess age and its variability in the literature, and most studies address wide age ranges. In contrast, in our study, 6-year-old children were analyzed, which could be considered a limitation. It should also be considered that in recent years, more global detection tools for NDs have been developed, and knowledge has been expanded, which may increase their prevalence, among other factors. It is challenging to determine the actual prevalence of each disorder, although with direct assessments, as in our case, we believe that some of the margin of error is reduced.

It is not clear whether there are differences in the prevalences calculated among clinical, school, and population samples, since the results found in the different studies analyzed are usually similar or rather disparate and contradictory, without observed patterns to establish a clear relationship. An example includes the study of Galicia [11], which included a clinical sample, paradoxically, with lower risks of presenting dyslexia (3.26%) or ADHD (5.35%) detected than those in other studies. In contrast, in a Norwegian study [9], which also included a clinical sample and direct assessments, prevalence rates similar to those obtained in our work were detected; in our study, 55.4% of the sample suffering from one or more NDs. A study in Japan [28] obtained figures for the prevalence of ASD similar to those calculated in our work (3.22% compared to 2.8%, respectively). Age did not seem to be an influencing factor in the rest of the studies and disorders. More comparative studies between population and clinical samples should be carried out to assess whether there is variability among different populations.

As a limitation of our work, it was difficult to delve into each disorder since the literature available for each disorder is very extensive, a fact that led us to generalize and synthesize results globally. It should be emphasized that we had an age limitation for learning problems since we could not make a diagnosis at 6 years of age, but we could provide early detection and subsequent follow-up. Our study was also limited in terms of psychomotor measures since, due to time constraints, we did not use clinical assessment instruments. Highlight the importance of considering gender differences in research, where the literature is closely linked to masculinity [51], which it considers more studies of equal proportion must be carried out to better define. In any case, in our sample, the proportion of men and women was similar. Because Menorca is an island with few private services, underdetection of NDs in girls may have occurred. Another limitation that should be highlighted is the size of the sample. Since this study included a population-based sample, which was small compared to other existing prevalence studies, prevalence estimates are less reliable (made through approximations by databases or health system registries). It should be considered that in our work, each child was evaluated by a trained professional using a direct assessment and that the risks detected would have a minimal margin of error. One drawback is that it is extremely expensive to carry out population-based studies with direct assessments, which is why prevalence studies generally resort to more imprecise estimates and larger sample sizes.

In addition, of the tests used to screen for the risk of presenting an ND, we used a recently developed test, the PROLEXIA battery, concluding that it seems to be a strong predictor of comorbidity and could be useful to determine other types of disorders associated with neurodevelopment. It should be added that other types of disorders that are not directly determined by the PROLEXIA tool include those that could contribute to this test, such as communication disorders or borderline cognitive levels, among others. We should consider a global test to determine the prevalence risk of different NDs. More research is needed to refine the prediction of what disorders this tool can predict apart from dyslexia.

## Conclusions

We consider that the island of Menorca fulfills an indispensable requirement for epidemiological studies, which is the stability of the population over time. Considering the importance of the study of prevalence in child and adolescent psychopathology carried out on the Isle of Wight in 1976 by Rutter et al. [52], we believe that we reached a real approximation of the population of the island and thus are able to compare, share and replicate the results within the scientific community.

Our work defends the importance of direct evaluation of children and their families by trained professionals to extract prevalence figures as close to reality as possible. In this sense, the sample studied was population-based, avoiding the biases that would derive from the study of a clinical sample. There are few studies that have directly evaluated individuals and their families, and this is the first study worldwide to evaluate children at an early age (6 years) from a sample recruited from primary care services. Important point about our sample are that it was a poorly selected sample, it represented the risk group for NDs in Menorca and it contrasted with the results of clinical samples.

The results obtained highlight the importance of early detection, the establishment of specific programs aimed at early detection and the training of professionals who can perform specific evaluations due to the increased demand for pediatric consultations for neurodevelopmental difficulties. Despite the increase in the number of requests for primary care pediatric consultations, the presence of an early concern of parents about their children’s language delays and other neurodevelopmental difficulties that has not been previously attended, detected, or studied by professionals is noteworthy. The study provides the following advantages: ecological validity in the area studied, emphasis on improvements in health planning, coordination between primary and specialized care services and improved early detection of subtle signs of NDs.

The existence of an evident connection between comorbidity and the involvement of these cases in populations with greater socioeconomic disadvantage and the need to provide assistance to these families is also important.

In conclusion, we recommend promoting care from the earliest stages of development, i.e., preconception, pregnancy and the first years of life, to minimize possible risks and thus model epigenetically. Moreover, the promotion of healthy lifestyle habits, such as consuming a healthy diet, practicing sports, and reducing the use of new technologies from the age of 3 years, should be emphasized. Likewise, NDs will affect the subjects and their families throughout their lives, so these disorders should be considered to reduce school failure rates and, consequently, the costs to education, society, families and children’s self-esteem.

## Data Availability

The data and materials are accessible from the main author. Correspondence about the manuscript should be addressed to Dr. Lorena Francés-Soriano.

## Abbreviations

ND: Neurodevelopmental disorder
ID: Intellectual disability
ADHD: Attention-deficit/hyperactivity disorder
ASD: Autism spectrum disorder
SLD: Specific learning disorder (e.g., dyslexia)
CD: Communication disorder
MD: Motor disorder
TS: Tourette’s syndrome
TD: Tic disorder
DCD: Developmental coordination disorder
DD: Developmental dyslexia
DLD: Developmental language disorder
SLI: Specific language impairment
DSM-5: Diagnostic and Statistical Manual of Mental Disorders, 5th Edition
ICD-10: International Statistical Classification of Diseases and Related Health Problems, 10th Revision
ICD-11: International Statistical Classification of Diseases and Related Health Problems, 11th Revision
WHO: World Health Organization
APA: American Psychiatric Association
